# Bezafibrate Attenuates Pressure Overload-Induced Cardiac Hypertrophy and Fibrosis

**DOI:** 10.1155/2017/5789714

**Published:** 2017-01-03

**Authors:** Si-Chi Xu, Zhen-Guo Ma, Wen-Ying Wei, Yu-Pei Yuan, Qi-Zhu Tang

**Affiliations:** Department of Cardiology, Renmin Hospital of Wuhan University, Cardiovascular Research Institute of Wuhan University, Wuhan, China

## Abstract

*Background.* Peroxisome proliferator-activated receptor-*α* (PPAR-*α*) is closely associated with the development of cardiac hypertrophy. Previous studies have indicated that bezafibrate (BZA), a PPAR-*α* agonist, could attenuate insulin resistance and obesity. This study was designed to determine whether BZA could protect against pressure overload-induced cardiac hypertrophy.* Methods.* Mice were orally given BZA (100 mg/kg) for 7 weeks beginning 1 week after aortic banding (AB) surgery. Cardiac hypertrophy was assessed based on echocardiographic, histological, and molecular aspects. Moreover, neonatal rat ventricular cardiomyocytes (NRVMs) were used to investigate the effects of BZA on the cardiomyocyte hypertrophic response in vitro.* Results.* Our study demonstrated that BZA could alleviate cardiac hypertrophy and fibrosis in mice subjected to AB surgery. BZA treatment also reduced the phosphorylation of protein kinase B (AKT)/glycogen synthase kinase-3*β* (GSK3*β*) and mitogen-activated protein kinases (MAPKs). BZA suppressed phenylephrine- (PE-) induced hypertrophy of cardiomyocyte in vitro. The protective effects of BZA were abolished by the treatment of the PPAR-*α* antagonist in vitro.* Conclusions.* BZA could attenuate pressure overload-induced cardiac hypertrophy and fibrosis.

## 1. Introduction

Cardiac hypertrophy is defined as an increase in cardiomyocyte size, interstitial fibrosis, and cardiac dysfunction [1, 2]. Cardiac hypertrophy can lead to ventricular arrhythmias and increase the incidence of fatal cardiovascular events [3]. The precise mechanisms regulating cardiac hypertrophy remain unclear. However, accumulating evidence indicates that protein kinase B (AKT)/glycogen synthase kinase-3*β* (GSK3*β*) and mitogen-activated protein kinases (MAPKs) play key roles in the development of cardiac hypertrophy [4].

AKT was activated in the heart after hypertrophic stimuli. Moreover, AKT overexpression induced a remarkable increase in cardiomyocyte cell size [5, 6]. AKT also resulted in the inactivation of GSK-3*β* and contributed to the process of cardiac hypertrophy [7, 8]. MAPKs were also closely associated with the development of the hypertrophic response. It has been reported that extracellular signal-regulated kinase (ERK) and P38 are activated in hypertrophic hearts and that inhibition of the activation of ERK and P38 might alleviate the hypertrophic response [9–11]. Therefore, finding drugs that can inhibit these prohypertrophic signaling pathways is of great importance.

Peroxisome proliferator-activated receptors (PPARs) are the nuclear receptor superfamily of ligand-activated transcription factors [12]. PPAR-*α*, which is highly expressed in the heart, could regulate the homeostasis of lipid metabolism [13, 14]. Previous studies have found that cardiac PPAR-*α* deficiency results in myosin dysfunction, with a pronounced decrease in cardiac contractile function and an increase in oxidative damage [15, 16]. Bezafibrate (BZA), a PPAR-*α* agonist, has been used widely in the treatment of hyperlipidemia and could also attenuate hepatic steatosis and modulate insulin resistance and obesity [17]. Moreover, results of previous research have indicated that the PPAR-*α* agonist suppressed the activation of AKT in noncardiomyocytes [18]. However, whether BZA can affect cardiac hypertrophy has not been clearly studied. This study was designed to investigate the effects of BZA on cardiac hypertrophy induced by pressure overload as well as to reveal the underlying mechanisms.

## 2. Materials and Methods

All animal experimental procedures were approved by the Guidelines for the Care and Use of Laboratory Animals of the Chinese Animal Welfare Committee and the guidelines of Renmin Hospital.

### 2.1. Reagents

BZA was acquired from Sigma (B7273, purity > 98%). Phenylephrine (PE, P1240000) was obtained from Sigma-Aldrich. Anti-PPAR-*α* (sc-9000) and anti-PCNA (sc-7907) were purchased from Santa Cruz Biotechnology. The following first antibodies were purchased from Cell Signaling Technology: anti-AKT (#4691), anti-phospho-AKT (#4060), anti-GSK3*β* (#9315), anti-phospho-GSK3*β* (#9323P), anti-ERK (#4695), anti-phospho-ERK (#4370P), anti-P38 (#9212P), anti-phospho-P38 (#4511P), anti-AMPK*α* (#2603P), and anti-phospho-AMPK*α* (#2535). Anti-GAPDH (#ab8245), anticalcineurin (CaN) (#ab90540), and anti-NFAT1 (#ab2722) were obtained from ABCAM. Anti-*α*-actinin was acquired from Millipore. The secondary antibodies were purchased from LI-COR Biosciences. The PPAR-*α* antagonist (GW6471, G5045), PPAR-*β*/*δ* antagonist (GSK0660, G5797), and PPAR-*γ* antagonist (GW9662, M6191) were all purchased from Sigma-Aldrich. All other chemicals were of analytical grade.

### 2.2. Animals and Treatment

Male C57BL/6 mice (8–10 weeks old) were purchased from the Institute of Laboratory Animal Science, CAMS & PUMC (Beijing, China), and fed in an environment with controlled temperature and humidity. The mice had the full ability to freely access water and food in a 12 h light-dark cycle. After one week, all the animals were randomly divided into 4 groups: sham + vehicle, sham + BZA, AB + vehicle, and AB + BZA. The dose of BZA used in our study was determined according to a previous article [19]. Mice were given BZA dissolved in saline (100 mg/kg, 17:00 every day) for 7 weeks beginning 1 week after the AB surgery. Mice in the control group were subjected to the same volume of saline. Details of the AB surgery were described in a previous article [20]. After seven weeks of treatment, the echocardiographic examinations were performed. Then, all the animals were euthanized before their hearts were collected and weighed.

### 2.3. Echocardiography Analysis and Hemodynamics Detection

Echocardiographic parameters were obtained according to our previous article [21]. A MyLab 30CV (Esaote SpA, Genoa, Italy) equipped with a 10 MHz linear array ultrasound transducer was used. The left ventricular end-systolic diameter (LVSD) and end-diastolic diameter (LVDD) were detected at the papillary level in M-mode tracing with a sweep speed of 50 mm/s. To measure the changes in the hemodynamics parameters, a microtip catheter transducer (SPR-839, Millar Instruments, Houston, TX, USA) was inserted into the carotid artery until it was in the left ventricle to monitor the pressure signals and heart rate continuously with an ARIA pressure-volume conductance system [22].

### 2.4. Histological Analysis

Hearts were arrested in 10% KCL and fixed with 10% formalin. Then, they were embedded with paraffin and cut transversely. Haematoxylin-eosin (HE) and picrosirius red (PSR) techniques were used for histological analysis. After staining, we used a digital analysis system (Image-Pro Plus, version 6.0; Media Cybernetics, Bethesda, MD, USA) to evaluate the cross-sectional area (CSA) of the myocytes and the percentage of collagen. We outlined at least 200 myocytes in each group.

### 2.5. Western Blot Analysis

RIPA buffer was used to extract the protein from the hearts. Total and nucleus protein were extracted as previously described [23, 24]. The concentrations of the proteins were detected using the BCA Protein Assay Kit (cat. number 23227; Thermo Fisher Scientific, Waltham, MA, USA). Then, the proteins were fractionated on the 10% SDS-PAGE and transformed onto the PVDF membrane (cat. number IPFL00010; EMD Millipore, Billerica, MA, USA). Subsequently, they were incubated with different primary antibodies overnight prior to incubation with secondary antibodies for 1 h. Finally, the membranes were analyzed and quantified using the Odyssey Infrared Imaging System (LI-COR Biosciences, Lincoln, NE, USA).

### 2.6. Real-Time Polymerase Chain Reaction Analysis

The RNA was extracted from the frozen hearts via TRIzol (cat. number 15596026; Invitrogen Life Technologies, Carlsbad, CA, USA). The cDNA was synthesized from 1 *μ*g RNA from each group using the Prime Script RT Reagent Kit (cat. number RR047Q; TAKARA BIOTECHNOLOGY (DALIAN) CO, LTD). Quantitative analysis was conducted using the LightCycler 480 SYBER Green Master Mix (cat. number 04896866001; Roche Diagnostics GmbH). All details about the primers are presented in Table 1.

### 2.7. Cell Culture and Staining

Neonatal rat ventricular myocytes (NRVMs) were isolated as described previously [25]. The NRVMs were cultured with Dulbecco's Modified Eagle Medium/Nutrient Mixture F-12 Ham (DMEM/F12) with 10% fetal bovine serum (FBS) (GIBCO, 10099), 1% streptomycin (100 mg/ml; GIBCO, 15140), and penicillin (100 U/ml). We used bromodeoxyuridine (0.1 mM) to prevent fibroblast growth. The purity of the cardiac myocytes was assessed by positive staining with antibodies *α*-actinin. The cells were first seeded onto six-well culture plates for 48 h with both DMEM and 10% FBS and then only supplied with 0.5% DMEM for 12 h. Finally, phenylephrine (50 *μ*mol) was added to the media to stimulate the cell with and without the BZA. Immunofluorescence staining was used to analyze the myocyte surface. To stain the cells, the NRVMs were fixed with 4% formaldehyde and infiltrated with 0.1% Triton X-100. Subsequently, the cells were stained with anti-*α*-actinin (1 : 100 dilution) before being incubated with Alexa Fluor 568-goat anti-mouse secondary antibody (Invitrogen, A11017). Image-Pro Plus 6.0 software was used to examine the cell areas.

### 2.8. Statistical Analysis

All data are expressed as the mean ± SD. The data were analyzed using one-way ANOVA. Tukey's test was used to conduct post hoc analyses. *P* < 0.05 was believed to indicate statistical significance.

## 3. Results

### 3.1. BZA Improved Cardiac Function in Mice Subjected to AB Surgery

**Figure 1:**
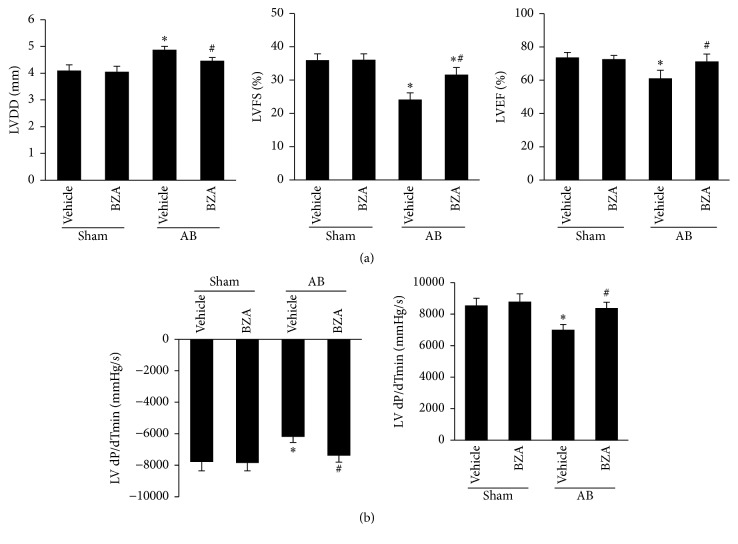
Echocardiographic and hemodynamic parameters in mice subjected to BZA (100 mg/kg). (a) Echocardiographic parameters (*n* = 10–13). (b) Hemodynamic parameters in the indicated groups (*n* = 8). Compared with sham + vehicle, ^*∗*^*P* < 0.05. Compared with AB + vehicle, ^#^*P* < 0.05.

The mice subjected to AB surgery developed deteriorated cardiac function, as evidenced by the increase in LVDD and the reduction in left ventricular fractional shortening (LVFS) and ejection fraction (LVEF) (Figure 1(a)). To investigate the effect of BZA, the mice were given BZA dissolved in saline (100 mg/kg, 17:00 every day) for 7 weeks beginning 1 week after AB surgery. These echocardiographic changes improved after BZA treatment. Pressure overload also resulted in a marked reduction in cardiac contractility, as measured by dP/dT max and dP/dT min (Figure 1(b)). BZA treatment restored impaired cardiac contractility (Figures 1(a)-1(b)).

### 3.2. BZA Attenuated Cardiac Hypertrophy Induced by AB Surgery

**Figure 2:**
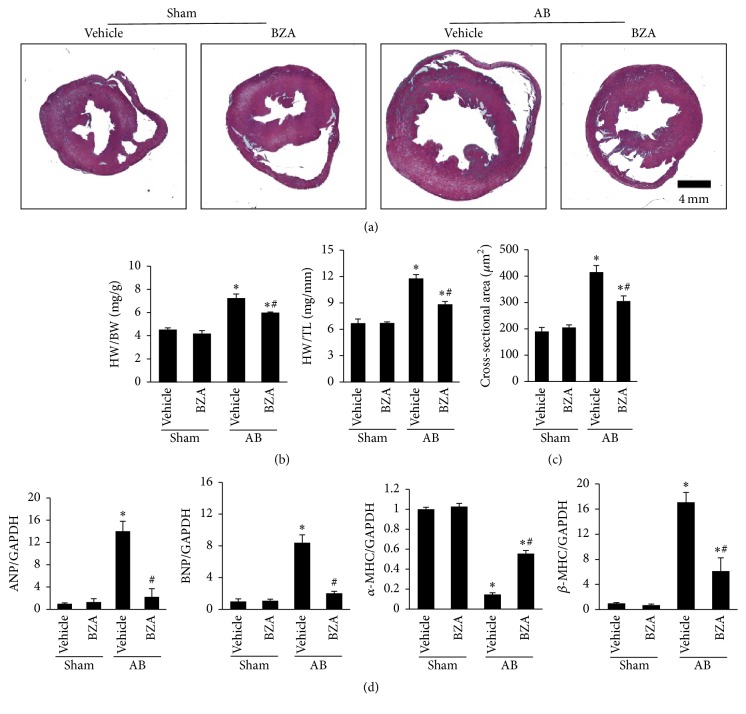
BZA suppressed cardiac hypertrophy in vivo. (a) Gross heart (*n* = 4). (b) The results of the HW/BW and HW/TL ratio (*n* = 12–15). (c) The cross-sectional areas in the sham and AB groups with and without BZA (*n* = 4). (d) The mRNA levels of ANP, BNP, *α*-MHC, and *β*-MHC (*n* = 6).

The mice subjected to AB surgery exhibited a significant hypertrophic response compared with the sham group, as illustrated by the increase in heart weight/body weight (HW/BW), heart weight/tibia length (HW/TL), and CSA (Figures 2(a)–2(c)). However, the hypertrophic response in the mice subjected to BZA treatment was significantly reduced. These results were corroborated by the analysis of atrial natriuretic peptide (ANP), brain natriuretic peptide (BNP), *α*-myosin heavy chain (*α*-MHC), and *β*-myosin heavy chain (*β*-MHC) (Figure 2(d)).

### 3.3. BZA Blocked Cardiac Fibrosis

**Figure 3:**
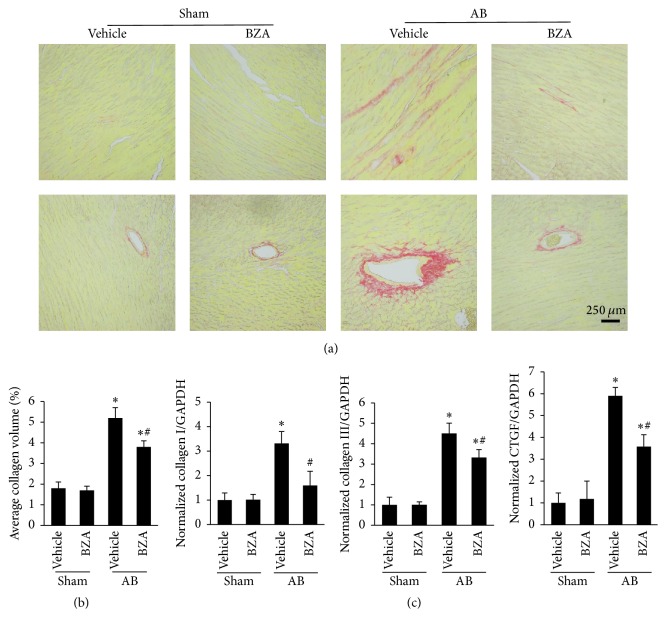
BZA suppressed cardiac fibrosis in vivo. (a) The histologic manifestation of PSR in both left ventricular and vascular fibrosis (*n* = 4). (b) The collagen level of the sham and AB groups in the presence and absence of BZA treatment (*n* = 4). (c) The mRNA level of collagen I, collagen III, and connective tissue growth factor (CTGF) (*n* = 6).

As observed in the mice subjected to AB surgery, the collagen deposition in both the interstitial and perivascular spaces increased (Figures 3(a)-3(b)). In addition, the mRNA levels of collagen I, collagen III, and connective tissue growth factor (CTGF) obviously increased in the AB group (Figure 3(c)). However, these pathological changes were alleviated after BZA treatment.

### 3.4. BZA Inhibited MAPKs and AKT/GSK3*β* Signal Pathways in Response to Hypertrophic Stimuli

**Figure 4:**
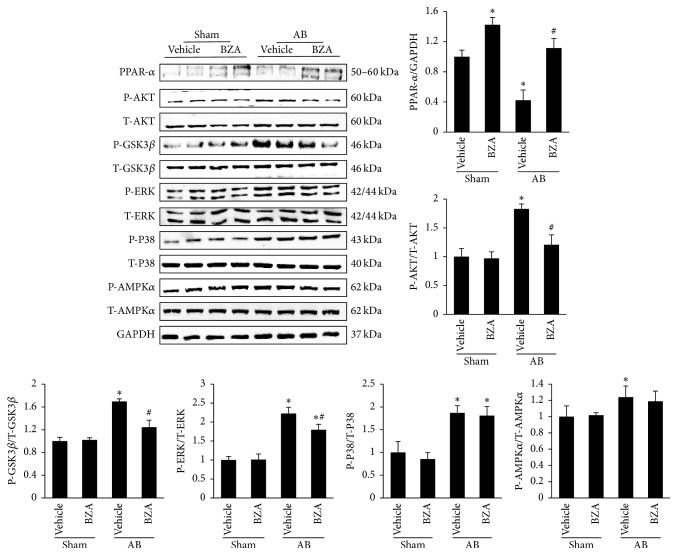
The effect of BZA on AKT/GSK3*β*, AMPK*α*, and MAPK signal pathways. The relative quantitative results of PPAR-*α*, P-AKT, P-GSK3*β*, P-ERK, P-P38, and P-AMPK*α* in the four groups (*n* = 6).

As shown in Figure 4, the protein level of PPAR-*α* was downregulated after AB surgery. Moreover, as a PPAR-*α* agonist, BZA could upregulate the decreased PPAR-*α*. Pressure overload resulted in the elevated phosphorylation of AKT and GSK3*β*. Conversely, BZA treatment suppressed the activated AKT/GSK3*β* pathway. In addition, PPAR-*α* activated by BZA could diminish the phosphorylation of ERK but not P-P38. There was no significant difference in P-AMPK*α* between the AB + vehicle and AB + BZA groups.

### 3.5. BZA Attenuated Cardiomyocyte Hypertrophy in the Presence of Phenylephrine In Vitro

**Figure 5:**
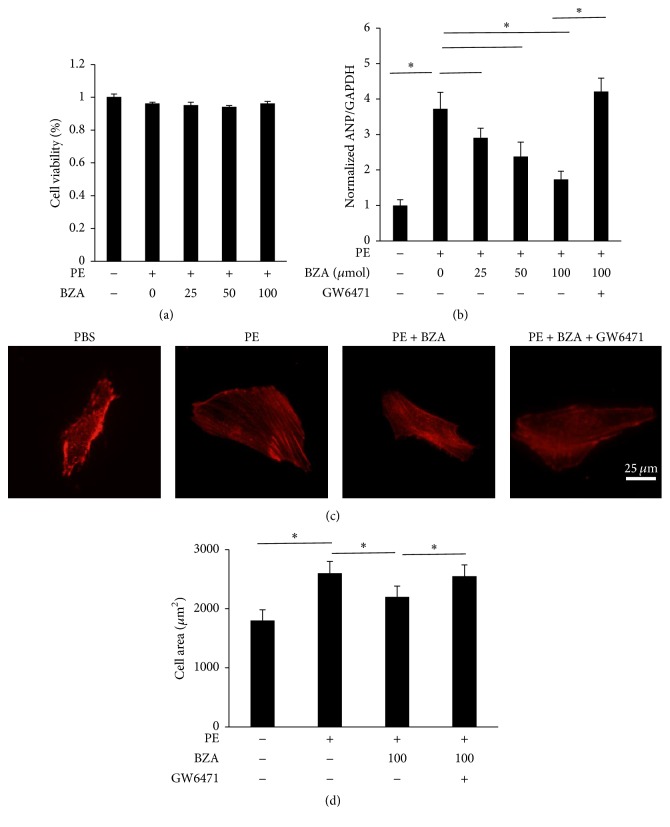
BZA blocked the process of cardiomyocyte hypertrophy in vitro. (a) The viability of neonatal rat ventricular cardiomyocytes stimulated by phenylephrine (*n* = 6). (b) The ANP levels in the presence of BZA and phenylephrine at different concentrations (*n* = 6). (c) The representative exhibition of a cardiomyocyte under immunofluorescence staining (*n* = 6). (d) The average cell area of cardiomyocytes (*n* = 6).

**Figure 6:**
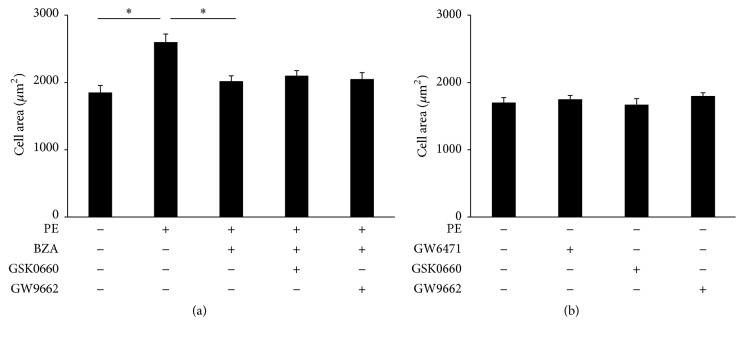
The effects of different PPAR isoforms antagonists under BZA treatment when stimulated with phenylephrine. (a) The average cell area of cardiomyocytes when treated with different kinds of PPAR isoforms with both phenylephrine and BZA (*n* = 6). (b) The average cell area of cardiomyocytes with a lone inhibitor (*n* = 6).

To further understand the effect of BZA on hypertrophy, NRVMs were subjected to PE to induce hypertrophy of myocytes in vitro (Figure 5). As expected, BZA decreased the increased cell areas and hypertrophic markers. Although the PPAR-*α* antagonist (GW6471, 20 *μ*mol) did not affect the cell area at baseline, GW6471 abolished the protection of BZA against hypertrophy, as indicated by the cell areas and ANP level (Figure 5). PPAR-*β*/*δ* (GSK0660, 1 *μ*mol) and PPAR-*γ* (GW9662, 10 *μ*mol) antagonists had no effect on the BZA-mediated protection (Figure 6).

### 3.6. BZA Had No Significant Effect on the CaN/NFAT-1 Signal Pathway, Inflammation, and Apoptosis

**Figure 7:**
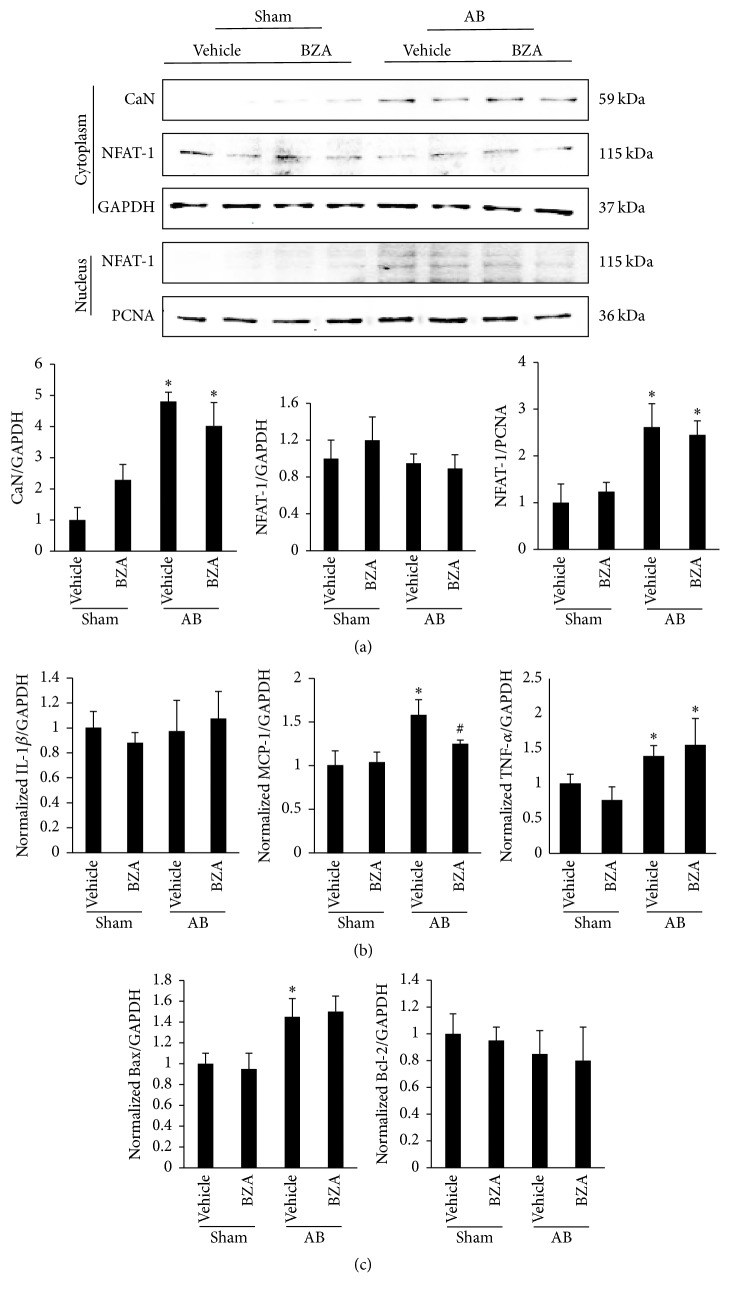
The effect of BZA on the CaN/NFAT1 signal pathway, inflammation, and apoptosis. (a) The protein level of CaN/NFAT1 in both cytoplasm and the nucleus (*n* = 6). (b) The mRNA level of IL-1*β*, MCP-1, and TNF-*α* under BZA treatment (*n* = 6). (c) The mRNA level of Bax and Bcl-2 (*n* = 6).

As shown in Figure 7, we measured the protein levels of the CaN/NFAT-1 signal pathway. There was no significant difference in CaN and NFAT-1 between the AB + vehicle and AB + BZA groups in terms of the cytoplasm and nucleus. Moreover, BZA also had no significant influence on the mRNA levels of Bax and Bcl-2. Regarding the inflammatory response, the mRNA levels of monocyte chemoattractant protein-1 (MCP-1) were slightly downregulated under BZA treatment, whereas interleukin-1*β* (IL-1*β*) and tumor necrosis factor-*α* (TNF-*α*) were not significantly affected by the BZA treatment.

## 4. Discussion

Our research demonstrated that BZA can inhibit cardiac hypertrophy in vivo and in vitro. BZA alleviated cardiac fibrosis induced by pressure overload. In addition, the phosphorylation of AKT/GSK*β* and MAPKs signal pathways were downregulated after BZA treatment. BZA also diminished PE-induced myocytes hypertrophy. The effects were abolished by the PPAR-*α* antagonist in vitro.

As an energy metabolic regulator, PPAR-*α* can modulate cardiac metabolism substrate conversion in cardiac hypertrophy, heart failure, and ischemic heart disease [26]. Results of previous research have illustrated that PPAR-*α* has various functions, including extracellular matrix remolding and the inflammatory response. Absence of PPAR-*α* resulted in a more pronounced hypertrophic response and deteriorated cardiac function accompanied by enhanced expression of markers of inflammation and extracellular matrix remodeling [27]. Activation of PPAR-*α* improved cardiac function in diabetic cardiomyopathy [28, 29]. PPAR-*α* agonists can block cardiac hypertrophy induced by endothelin-1 [30, 31]. Consistent with the findings of these studies, our results also demonstrated that BZA attenuates cardiac hypertrophy in vivo and in vitro. However, a divergent perspective was that mice with cardiac overexpression of PPAR-*α* developed spontaneous cardiomyocyte hypertrophy [32]. In previous research, the protein levels of PPAR-*α* were more abundant (15–135-fold) in the hearts of transgenic animals than in their nontransgenic littermates. In our study, PPAR-*α* was slightly activated by BZA, which may explain the discrepancies between different studies. Previous studies also reported that the effects of PPAR agonists on the heart are mediated by non-PPAR effects. Fenofibrate was found to exert deleterious pleiotropic myocardial actions in PPAR-*α*-deficient mice [33]. Inconsistent with the findings of previous studies, our study demonstrated that the protective effects of BZA on cardiomyocyte hypertrophy can be blocked by the PPAR-*α* antagonist rather than the PPAR*β*/*δ* and PPAR-*γ* inhibitor, implying that BZA attenuates cardiac hypertrophy via PPAR-*α*. This finding suggests that the excessive rate of myocardial fatty acid uptake coupled with reduced glucose utilization may result in excessive lipid accumulation in the heart and exaggerated cardiac remolding. There are reasons to believe that BZA treatment simply recovers the energy balance to the normal condition, which prevents the progression of cardiac hypertrophy.

Previous research has illustrated that the overexpression of AKT results in obvious cardiac hypertrophy, with a significant increase in cardiomyocyte size [34]. Moreover, the hypertrophic response can be alleviated in AKT knockout mice [35]. The results of our lab work indicate that inhibition of AKT/GSK3*β* obviously attenuates pressure overload-induced cardiac hypertrophy [36]. Fenofibrate, a PPAR-*α* agonist, was previously found to alleviate renal ischemia-infusion injury and glucose-induced matrix deposition via the AKT pathway [37, 38]. Moreover, fenofibrate alleviated endothelin-1-induced cardiomyocyte hypertrophy by inhibiting the phosphorylation of AKT and GSK3*β* [39]. Consistent with the findings of these studies, our data also revealed that AKT/GSK3*β* were downregulated under BZA treatment, implying that AKT/GSK3*β* played a role in the cardioprotection mediated by BZA. ERK was activated in response to hypertrophic stimuli in neonatal cardiomyocytes. Moreover, the pharmacological inhibition of ERK significantly impeded the hypertrophic response [40]. It has been reported that PPAR-*α* activation could ameliorate aldosterone-induced cardiac remodeling in adult rat ventricular myocytes, partly by inhibiting the phosphorylation of ERK [41]. In our study, the phosphorylation of ERK was significantly downregulated with BZA treatment after AB surgery. P38 was also closely associated with cardiac hypertrophy [42]. The specific P38 inhibitor or negative P38 mutant expression attenuated cardiomyocyte growth in response to hypertrophic stimuli, whereas overexpression of P38 resulted in a hypertrophic response [43, 44]. Previous research indicated that the activation of PPAR-*α* reduced P-P38 to alleviate renal injury [45]. However, a divergent perspective was that PPAR-*α* suppressed melanogenesis via upregulation of P-P38 [46]. Inconsistent with the findings of these studies, our results indicated that BZA had no obvious effect on the level of P-P38. The reason for the inconsistent results is that P38 plays different roles in different pathological processes.

Acting as an energy sensor, AMPK*α* has been most widely investigated in energy metabolism in both physiological and pathological conditions [47]. Our previous work demonstrated that activation of AMPK*α* can alleviate the hypertrophic response [48, 49]. Moreover, it has been shown that PPAR-*α* can enhance the phosphorylation of AMPK*α* to reduce reticulum stress induced by the palmitate in human cardiac cells [50]. Unexpectedly, no significant difference in AMPK*α* was observed after BZA treatment in the AB group in our study, implying that AMPK*α* does not contribute to the protective role of BZA in cardiac hypertrophy.

Previous research has demonstrated that CaN-NFAT plays a significant role in the development of cardiac hypertrophy and that overactivation of PPAR-*α* might inhibit the nuclear translocation of NFAT-1 from the cytoplasm to the nucleus [39, 51]. However, our study discovered that there was no significant difference in the protein level of CaN/NFAT-1 between the AB + vehicle and AB + BZA groups. Inflammatory biomarkers were upregulated under the hypertrophic response. Early studies had discovered that activation of PPAR-*α* could inhibit inflammatory activation and reduce the activity of macrophages in the development of atherosclerosis [52, 53]. Our results revealed that only MCP-1 was slightly downregulated under BZA treatment. In addition, cell apoptosis was detected in our study. The mRNA levels of both Bax and Bcl-2 remained approximately unchanged under BZA treatment. All of these results imply that the protective effect of BZA was not mediated by CaN/NFAT1, inflammation, and apoptosis pathways.

Fibrosis, another crucial pathophysiological process in cardiac remolding, is characterized by the accumulation of collagen and the deposition of extracellular matrix. A previous study demonstrated that PPAR-*α* KO mice exhibit progressive cardiac fibrosis and aggravated cardiac hypertrophy [50]. Chen et al. [54] found that activation of PPAR-*α* attenuated liver fibrosis induced by a methionine choline-deficient diet. Moreover, Suk et al. [55] demonstrated that BZA inhibits fibrogenesis in a murine steatohepatitis model. Consistent with the findings of these studies, we observed that BZA treatment attenuates AB-induced cardiac fibrosis. The activation of PPAR-*α* caused by BZA treatment may be the underlying mechanism that mediates antifibrotic effects.

In conclusion, our study demonstrated that BZA can protect against cardiac hypertrophy induced by pressure overload. BZA also suppressed the activation of AKT/GSK3*β* and ERK in hypertrophic hearts. BZA alleviated PE-induced hypertrophy of cardiomyocytes via PPAR-*α*. Our study provides experimental evidence for the application of BZA in the treatment of cardiac hypertrophy.

## Figures and Tables

**Table 1:** Primers used in the study.

Gene	Species		Sequence (5′-3′)
GAPDH	Mouse	Forward	ACTCCACTCACGGCAAATTC
		Reverse	TCTCCATGGTGGTGAAGACA
ANP	Mouse	Forward	ACCTGCTAGACCACCTGGAG
		Reverse	CCTTGGCTGTTATCTTCGGTACCGG
*α*-MHC	Mouse	Forward	GTCCAAGTTCCGCAAGGT
		Reverse	AGGGTCTGCTGGAGAGGTTA
*β*-MHC	Mouse	Forward	CCGAGTCCCAGGTCAACAA
		Reverse	CTTCACGGGCACCCTTGGA
BNP	Mouse	Forward	GAGGTCACTCCTATCCTCTGG
		Reverse	GCCATTTCCTCCGACTTTTCTC
Collagen I	Mouse	Forward	TGGTACATCAGCCCGAAC
		Reverse	GTCAGCTGGATAGCGACA
Collagen III	Mouse	Forward	GTCAGCTGGATAGCGACA
		Reverse	GAAGCACAGGAGCAGGTGTAGA
CTGF	Mouse	Forward	TGTGTGATGAGCCCAAGGAC
		Reverse	AGTTGGCTCGCATCATAGTTG
IL-1*β*	Mouse	Forward	CCGTGGACCTTCCAGGATGA
		Reverse	GGGAACGTCACACACCAGCA
MCP-1	Mouse	Forward	TGGCTCAGCCAGATGCAGT
		Reverse	CCAGCCTACTCATTGGGATCA
TNF-*α*	Mouse	Forward	CATCTTCTCAAAACTCGAGTGACAA
		Reverse	TGGGAGTAGATAAGGTACAGCCC
Bax	Mouse	Forward	TGAGCGAGTGTCTCCGGCGAAT
		Reverse	GCACTTTAGTGCACAGGGCCTTG
Bcl-2	Mouse	Forward	TGGTGGACAACATCGCCCTGTG
		Reverse	GGTCGCATGCTGGGGCCATATA
